# First Experience with the Nimbus Stentretriever: A Novel Device to Handle Fibrin-rich Clots

**DOI:** 10.1007/s00062-022-01237-z

**Published:** 2022-12-02

**Authors:** Nils C. Lehnen, Daniel Paech, Stefan Zülow, Felix J. Bode, Gabor C. Petzold, Alexander Radbruch, Franziska Dorn

**Affiliations:** 1 Department of Neuroradiology, University Hospital Bonn, Rheinische Friedrich-Wilhelms-Universität Bonn, 53127 Bonn, Germany; 2 Division of Vascular Neurology, Department of Neurology, University Hospital Bonn, Rheinische Friedrich-Wilhelms-Universität Bonn, 53127 Bonn, Germany

**Keywords:** Mechanical thrombectomy, Stroke, Reperfusion, Endovascular treatment, Second line device

## Abstract

**Purpose:**

To share our first experience with the Nimbus stentretriever, a multizone device designed to assist neurointerventionalists in handling fibrin-rich clots in endovascular stroke treatment.

**Methods:**

We retrospectively analyzed the data of patients who were treated with the Nimbus stentretriever at our high-volume stroke center between May 2021 and May 2022. We evaluated the number of passes before Nimbus was used, the number of passes with nimbus, as well as the recanalization success before and after Nimbus according to the modified treatment in cerebral ischemia (mTICI) scale. Also, patient characteristics, procedural times and clinical outcomes were documented.

**Results:**

A total of 21 consecutive patients were included in the study. An mTICI 2b/3 could be achieved in 76.2% and mTICI 2c/3 could be achieved in 57.1%. The mean number of passes was 3.4 before the use of Nimbus, 2.2 with Nimbus, and 5.4 for all passes with and without Nimbus and 4 occlusions (19.0%) were successfully recanalized with direct aspiration after the use of Nimbus. We observed seven subarachnoid hemorrhages (33.3%) and two cases of vasospasm.

**Conclusion:**

In our series, the use of Nimbus resulted in successful recanalization in half of the patients after otherwise unsuccessful thrombectomy maneuvers; therefore, it should be considered as a rescue option if the maneuver with conventional stent retrievers was unsuccessful.

## Introduction

Mechanical thrombectomy (MTE) has become the standard of care for patients with ischemic stroke and large vessel occlusion (LVO). The HERMES collaboration reported successful reperfusion rates with mTICI 2b‑3 of 71% [1]. Although devices and experience of neurointerventionalists have improved over the years, there still remain 20–30% of cases where sufficient reperfusion cannot be achieved [2]. The rate of successful reperfusion has been reported as high as 83% in the German Stroke Registry, a real-life multicentric thrombectomy registry [3]. The most common reason for unsuccessful thrombectomy maneuvers is the inability of thrombus removal, even with multiple stentretriever and/or aspiration passes [4]. On the other hand, it is well known that the likelihood of a successful recanalization decreases with every pass [5], presumably due to a condensation of the thrombus and thereby an increased fibrin-rich portion of the thrombus; this results in a sticky and even harder to remove clot [6]. Furthermore, the risk for symptomatic intracranial hemorrhage (sICH) increases from the fourth pass on [7]. Nimbus (Cerenovus, Johnson & Johnson, New Brunswick, NJ, USA) is a geometric clot extractor and has been designed to retrieve especially fibrin-rich clots by combining a proximal spiral section, and a distal classical stentretriever section. The two parts can be distinguished by their radiopaque markers. The device can be delivered through a standard microcatheter with an inner diameter of 0.021″ or 0.027″. On the benchmark, it has been proven to be able to remove fibrin-rich clots more efficiently than the Solitaire stent retriever (Medtronic, Minneapolis, MN, USA) [8]. We hereby report the performance of Nimbus as a second line device for MTE in LVOs at our hospital.

## Material and Methods

Institutional Review Board approval was obtained for this retrospective study and the need for written informed consent was waived.

This retrospective analysis was performed using patient data from the database of our high-volume stroke center between June 2021 and May 2022. At our institution, MTE is performed by four trained neurointerventionalists. The selection for i.v. lysis and endovascular stroke treatment was made according to national and international stroke guidelines.

Baseline patient characteristics as well as their clinical outcome were taken from the electronic medical records. Procedural parameters, such as the number of passes and mTICI scores as well as complications, were taken from the written report or, when not documented in the written report, from the image documentation in our PACS. Classification of intracranial hemorrhage was performed according to the Heidelberg bleeding classification [9].

Our general approach to MTE in the anterior circulation is to use a short 8F sheath and an 8F balloon guiding catheter which is placed at the cervical portion of the internal carotid artery. Then, a 5F intermediate catheter is placed as close as possible to the thrombus, a microcatheter and a microwire are introduced to pass the thrombus, and a stentretriever is placed at the level of the thrombus. The intermediate catheter is adapted to the thrombus and with the balloon inflated, both the intermediate catheter and the stentretriever are removed with permanent manual aspiration both at the balloon guiding catheter and the aspiration catheter. Apart from Nimbus, the following stentretrieval devices were used: Solitaire X (Medtronic), APERIO (Acandis, Pforzheim, Germany), Embotrap (Cerenovus, Johnson & Johnson), and Trevo NXT (Stryker, Kalamazoo, MI, USA). The intermediate catheter was 5F Sofia (Microvention, Tustin, CA, USA). The choice of material for the intervention, the decision to use Nimbus as well as the decision to continue or end the procedure were at the discretion of the interventionalist.

**Fig. 1:**
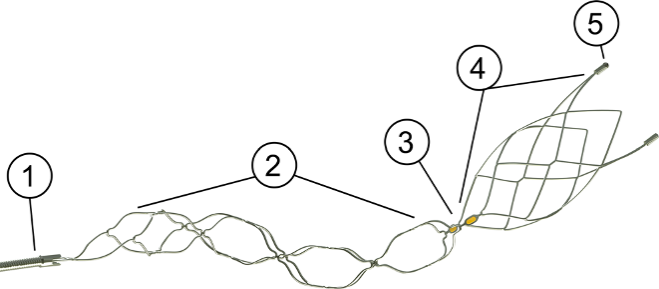
Nimbus and its different sections. 1 proximal radiopaque coil, 2 spiral section, 3 two mid-markers, 4 barrel section, 5 two distal markers. The working length of the device is 28 mm, the distal outer cage diameter 4.5 mm. Image copyright: Cerenovus, legends and dimensions added by the authors according to the instructions for use

**Fig. 2:**
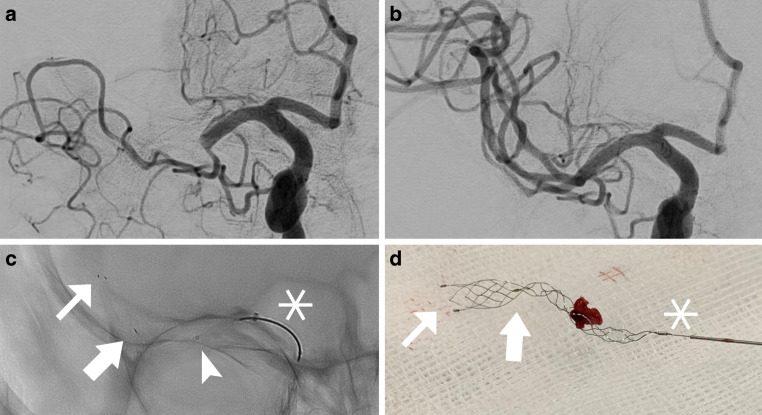
Occlusion of the M1 segment of the right middle cerebral artery. Angiographic images **a** before and **b** after one pass with Nimbus (mTICI 3). Prior to the use of Nimbus, two passes with Embotrap 5/37 mm were performed without recanalization. **c** Image of Nimbus deployed in the middle cerebral artery; the distal, barrel-like section of the device is situated between the two distal radiopaque markers (*small arrows* in **c** and **d**) and the proximal marker (*large arrows* in **c** and **d**). The proximal, spiral-like part of the device is indicated by the proximal radiopaque marker and the proximal, radiopaque coil (*asterisk*). The device is placed with the spiral section at the level of the thrombus, the microcatheter (tip indicated by *arrowhead* in **c**) has already been readvanced partially over the spiral part of the device (pinching), a resistance was felt and both the microcatheter and the device were removed. **d** Nimbus with clot after a successful thrombectomy maneuver

The use of Nimbus differs from other stentretrievers and all interventionalists underwent a training with a flow model with Nimbus before using the device in a clinical setting. The proximal, spiral part must be placed at the level of the thrombus. After passively deploying the device, the microcatheter is readvanced to the spiral part to pinch the clot, until the interventionalist feels a resistance. By readvancing the microcatheter over the spiral section of Nimbus (pinching maneuver), force is applied to the cells of the spiral section; subsequently, the cells close and thereby grip the clot (microclamping). Now, the interventionalist can remove both the microcatheter and Nimbus while maintaining the pinch. If an intermediate catheter is used, the authors were advised by the vendor to build up aspiration force after the pinching has been achieved, to prevent movement of the thrombus before pinching. Nimbus can either be pulled through the aspiration catheter, or it can be removed with the aspiration catheter, the latter being the method of choice at our institution to prevent thrombotic material from shearing off the tip of the aspiration catheter. A detailed description of the sections of Nimbus is shown in Fig. 1. The positioning of Nimbus is illustrated in Fig. 2.

Statistical analysis was performed using R version 4.0.3/R Foundation for Statistical Computing, Vienna, Austria) and RStudio version 1.2.5033 (RStudio Inc., Boston, MA, USA). For determining statistical significance, the Mann-Whitney U‑test was used.

## Results

### Baseline Characteristics

Between June 2021 and May 2022 a total of 170 stroke thrombectomies were performed at our center. In 28 patients, sufficient recanalization was not reached; in 21 of these 28 patients Nimbus was used after futile attempts with standard stentretrievers. Of the 21 patients, 13 (61.9%) were female, 8 patients were male. Mean age was 76.9 years (median 77 years, range 50–93 years) and 3 patients (14.3%) received i.v. lysis. The occlusion site was the M1 segment of the middle cerebral artery in 15 patients (71.4%) and the M2 segment in 6 patients. The majority of strokes were caused by atrial fibrillation (11 patients, 52.4%), 5, (23.8%) were caused by arterio-arterial embolization, 1 (4.8%) was due to an embolic complication during an endovascular aortic valve implantation and stroke origin was unknown in 4 stroke patients (19.0%). Pre-stroke mRS was 0–2 in 14 patients (66.7%) and 3–5 in 6 patients (28.6%). For one patient, pre-stroke mRS was not documented in the electronic medical record. Mean NIHSS score at presentation was 14 (median 14, range 4–24). Median ASPECTS was 9 (range 5–10).

Baseline characteristics are summarized in Table 1.

**Table 1:** Baseline characteristics of patients treated with Nimbus

Characteristics	n (%)
Overall	21 (100.0%)
Male	8 (38.1%)
Female	13 (61.9%)
Age (years, mean, range)	76.9 (50–93)
I.v. lysis	3 (14.3%)
Occlusion site	
M1	15 (71.4%)
M2	6 (28.6%)
Etiology of stroke	
Atrial fibrillation	11 (52.4%)
Arterio-arterial	5 (23.8%)
Iatrogenic	1 (4.8%)
Unknown/other	4 (19.0%)
NIHSS at admission	
0	0 (0.0%)
1–4	1 (4.8%)
5–15	10 (47.6%)
≥ 16	10 (47.6%)
Unknown	1 (4.8%)
Pre-stroke mRS	
0–2	14 (66.7%)
3–5	6 (28.6%)
Unknown	1 (4.8%)
ASPECTS (median, range)	9 (5–10)

*M1* M1 segment of the middle cerebral artery, *M2* M2 segment of the middle cerebral artery, *NIHSS* National Institutes of Health Stroke Scale, *mRS* modified Rankin Scale, *ASPECTS* Alberta stroke programme early CT score.

### Procedural Characteristics and Efficacy Outcomes

All patients were treated under general anesthesia. For 10 patients, the time of stroke onset was unknown. For the remaining 11 patients, the mean time from symptom onset to final reperfusion was 318 min (range 222–508 min). The mean time from groin puncture to reperfusion was 131 min (range 40–273 min) and 2 patients needed stenting of the cervical internal carotid artery. The mean number of passes before Nimbus was used was 3.4 (median 3, range 1–7). The mean number of passes with Nimbus was 2.2 (median 2, range 1–5). The mean total number of passes was 5.9 (median 6, range 3–10). Before the use of Nimbus, mTICI 2b could be achieved in 3 cases (14.3%), after 1 pass with Nimbus, mTICI 2b/3 was achieved in 8 patients (38.1%), and after all passes with Nimbus, mTICI 2b/3 was achieved in 12 patients (57.1%). An mTICI 2c/3 could be achieved in 4 patients after the first pass with Nimbus (19.0%), and after all passes with Nimbus, mTICI 2c/3 was achieved in 8 patients (38.1%). In another 4 patients, mTICI 2b/3 could be achieved with aspiration thrombectomy without the use of another stentretriever after the futile use of Nimbus, with a final mTICI 2b/3 in 16 patients (76.2%) and a final mTICI 2c/3 in 12 patients (57.1%). Among the mTICI 2b/3 group, the average number of passes with Nimbus was 2.1 (range 1–5).

### Complications and Clinical Outcome

We observed circumscribed subarachnoid hemorrhage (SAH) in seven patients (33.3%), of which one was identified as symptomatic. No patient underwent neurosurgical intervention due to intracranial hemorrhage but one underwent hemicraniectomy due to malignant edema. Vasospasm could be observed in two cases immediately after Nimbus passage (9.5%) and did not require further intervention in both of these cases, 4 patients (19.0%) achieved an mRS of 0–2 at discharge, 11 (52.4%) were discharged with an mRS of 3–5, and 6 patients (28.6%) died during hospital stay. Among the survivors, the mean improvement of NIHSS score was 3.1 (median 2, range −10–21). One patient (4.8%) experienced a decline of more than four points on the NIHSS scale, while the remaining patients either experienced an improvement of at least four points on the NIHSS scale (*n* = 6, 28.6%) or remained unchanged (*n* = 14, 66.7%). The mean total number of passes in patients with SAH was 6.6 (median 8, range 3–9), the mean total number of passes in patients without SAH was 5.5 (median 5, range 3–10). We could not find a significant difference between the number of passes in patients with and without SAH (*p* = 0.24).

Procedural characteristics, complications and clinical outcome are summarized in Table 2.

**Table 2:** Interventional parameters and clinical outcome of patients treated with Nimbus

Characteristics	Value
*Treatment time (min.)*	318 (222–508)
*Intervention time (min.)*	131 (40–273)
*Number of passes*	
Before Nimbus	3.4 (1–7)
With Nimbus	2.2 (1–5)
Total	5.9 (3–10)
*mTICI 2b/3*	
Before Nimbus	3 (14.3%)
After first Nimbus	8 (38.1%)
After last Nimbus	12 (57.1%)
Final	16 (76.2%)
*mTICI 2c/3*	
Before Nimbus	0 (0.0%)
After first Nimbus	4 (19.0%)
After last Nimbus	8 (38.1%)
Final	12 (57.1%)
*Complications*	
SAH	7 (33.3%)
sICH	1 (4.8%)
Vasospasm	2 (9.5%)
*mRS at discharge*	
0–2	4 (19.0%)
3–5	11 (52.4%)
6	6 (28.6%)
*NIHSS at discharge*	
0	1 (4.8%)
1–4	4 (19.0%)
5–15	4 (19.0%)
≥ 16	6 (28.6%)
Unavailable	6 (28.6%)
*NIHSS improvement*	
Mean	3.1
Median	2
Range	−10–21

*mTICI* modified treatment in cerebral ischemia, *SAH* subarachnoid hemorrhage, *sICH* symptomatic intracranial hemorrhage, *mRS* modified Rankin scale.

## Discussion

In our single-center study we retrospectively analyzed our first 21 patients who were treated with a novel thrombectomy device due to ischemic stroke with LVO.

Our goal was to assess the safety and efficacy of the Nimbus stentretriever in our patient cohort, as well as to analyze the clinical outcome and the rate of complications. Nimbus can be used both by experienced and less experienced interventionalists, although a training in a flow model is recommended to get used to the specific technique of pinching and retrieving the device. With Nimbus, we were able to achieve mTICI 2b/3 recanalization in 76.2% of patients in whom recanalization failed with conventional techniques. In 57.1% of our patients, we were able to achieve mTICI 2c/3 at the end of the procedure. We observed SAH in 33% of cases, of which only one was categorized as symptomatic, probably mainly due to the overall high number of passes that were necessary in some cases. Maros et al. reported the rate of postinterventional ICH to be 14.3% in an analysis of the German Stroke Registry, which is lower than in our cohort. Nevertheless, they found the rate of sICH to be 4.4%, which is in line with our findings, despite our relatively high average number of passes [7]. Thus, we have no reason to suspect the rate of sICH to be higher than with any other stentretriever, although we cannot draw a final conclusion here due to the low number of patients in our study. Interestingly, in 4 out of 16 patients in whom recanalization was successful, the recanalization was not achieved with Nimbus, but with pure contact aspiration after the futile use of Nimbus. Right now, we regard Nimbus to be a second-line device in challenging thrombectomy cases. Nimbus may be a suitable first-line device for fibrin-rich clots, however, it is not designed to retrieve red blood cell rich clots. Although progress has been made in the prediction of clot composition, either by imaging with the use of machine learning algorithms [10] or endovascularly by the use of electrical impedance spectroscopy [11], no reliable method to predict thrombus composition has been introduced into clinical practice. Another aspect is that Nimbus is more expensive when compared to most conventional stentretrievers.

Although we were able to recanalize more than three quarters of the occlusions, only 19% of the patients achieved a favorable outcome with an mRS of 0–2. This may be due to the high number of passes before the use of Nimbus and the long procedure times in some cases. While in the beginning of our work with Nimbus, we tended to try other stentretrievers or contact aspiration alone after two or three futile attempts instead of switching to Nimbus, we now tend to use Nimbus as a second-line device after two futile maneuvers with standard stentretrievers as our experience with the device grows. Maros et al. found that more than three stentretriever passes were a strong predictor for sICH, thus we hope to reduce the risk of sICH in our patients by earlier use of Nimbus after two futile thrombectomy maneuvers with a standard stentretriever [7]. As our mean number of passes with Nimbus was 2.1 in patients who could achieve mTICI 2b/3, we suggest that at least two passes should be performed with Nimbus. If Nimbus also fails, aspiration only achieved successful recanalization in a high number of patients in our cohort, thus being a relatively simple and potential successful option before ending the procedure or performing a rescue stenting. We are aware that this approach is not supported by sufficient data and must be viewed as a local algorithm that can be adjusted to the interventionalist’s preferences and to the patient characteristics.

There have been other attempts to improve recanalization rates in cases that cannot be recanalized with the standard technique, like the simultaneous use of two stentretrievers [12–16]. Recanalization success of up to 80% has been described with this technique by Klisch et al. in a small cohort of 10 patients who could not be reperfused with a single stentretriever. Recently, Vega et al. even suggested the double stentretriever thrombectomy as a first-line technique in patients with M1 or distal carotid occlusions: in a cohort of 39 patients, they achieved mTICI 2b/3 in 100% of cases [17]. Also, there have been reports that a fast stent retrieval can improve recanalization rates for fibrin-rich clots in vitro [18] and may improve recanalization rates of TICI 2b or better in 90% of the cases with large vessel occlusion due to fibrin-rich clots [19]. There have also been introduced other new devices for mechanical thrombectomy, like the radially adjustable Tigertriever (Rapid Medical, Sunrise, FL, USA) with recanalization rates of mTICI 2b/3 of 95.7% [20], and large bore aspiration catheters with an inner lumen of 0.088″ like the Route 92 Medical Reperfusion System (Route 92 Medical Inc, San Mateo, CA, USA) with rates of mTICI 2b/3 of 96% [21] or the Millipede 0.088 (Perfuze Ltd, Galway, Ireland), with promising preclinical results [22]. For patients with intracranial atherosclerosis, it may be impossible to achieve a successful recanalization with any thrombectomy device and emergency intracranial stenting has been suggested as a rescue treatment [23], with significantly more favorable outcomes in patients who received intracranial stenting instead of no further treatment after failed mechanical thrombectomy [24].

There are limitations to our study. First, its retrospective nature without a control group does not allow us to draw conclusions on the efficacy and safety of Nimbus compared to other devices or approaches. Second, our patient cohort is small but is still the largest and, to the best of our knowledge, only patient cohort in the literature that has been treated with Nimbus as a second-line device. Third, Nimbus is designed to retrieve fibrin-rich clots, but we have not performed a histologic analysis on thrombus composition in this retrospective study but assume that clots that cannot be retrieved with standard techniques are very likely fibrin-rich clots. Fourth, we only used 5F aspiration catheters, as it is our standard approach to MTE to use a balloon guiding catheter, making it impossible to use a 6F aspiration catheter, an ongoing discussion that is beyond the scope of our study.

## Conclusion

While rates of successful recanalization have improved over the years, there is still a subgroup of patients in whom recanalization cannot be achieved with standard techniques. Nimbus might be a promising device that might be used as an alternative to improve recanalization rates after otherwise failed thrombectomy and it may become a potential first-line device as soon as reliable methods are available to predict thrombus composition in the clinical routine. Although mostly clinically insignificant, we found a comparably high rate of postinterventional subarachnoid hemorrhage in our collective that needs to be further investigated in prospective registries and studies with larger patient cohorts.

## Abbreviations

ASPECTS: Alberta stroke programme early CT score
LVO: Large vessel occlusion
mRS: Modified Ranking scale
MTE: Mechanical thrombectomy
mTICI: Modified treatment in cerebral ischemia
NIHSS: National Institutes of Health Stroke Scale
PACS: Picture archiving and communication system
SAH: Subarachnoid hemorrhage
sICH: Symptomatic intracranial hemorrhage
